# A framework for exploring non-response patterns over time in health surveys

**DOI:** 10.1186/s12874-021-01221-0

**Published:** 2021-02-18

**Authors:** Famke J. M. Mölenberg, Chris de Vries, Alex Burdorf, Frank J. van Lenthe

**Affiliations:** 1 Department of Public Health, Erasmus MC, University Medical Center Rotterdam, P.O. Box 2040, 3000 CA Rotterdam, The Netherlands; 2 Department of Research and Business Intelligence, Municipality of Rotterdam, Rotterdam, The Netherlands

**Keywords:** Epidemiological methods, Response, Non-response bias, Surveys and questionnaires, Health surveys

## Abstract

**Background:**

Most health surveys have experienced a decline in response rates. A structured approach to evaluate whether a decreasing - and potentially more selective - response over time biased estimated trends in health behaviours is lacking. We developed a framework to explore the role of differential non-response over time. This framework was applied to a repeated cross-sectional survey in which the response rate gradually declined.

**Methods:**

We used data from a survey conducted biannually between 1995 and 2017 in the city of Rotterdam, The Netherlands. Information on the sociodemographic determinants of age, sex, and ethnicity was available for respondents and non-respondents. The main outcome measures of prevalence of sport participation and watching TV were only available for respondents. The framework consisted of four steps: 1) investigating the sociodemographic determinants of responding to the survey and the difference in response over time between sociodemographic groups; 2) estimating variation in health behaviour over time; 3) comparing weighted and unweighted prevalence estimates of health behaviour over time; and 4) comparing associations between sociodemographic determinants and health behaviour over time.

**Results:**

The overall response rate per survey declined from 47% in 1995 to 15% in 2017. The probability of responding was higher among older people, females, and those with a Western background. The response rate declined in all subgroups, and a faster decline was observed among younger persons and those with a non-Western ethnicity as compared to older persons and those with a Western ethnicity. Variation in health behaviours remained constant. Prevalence estimates and associations did not follow the changes in response over time. On the contrary, the difference in probability of participating in sport gradually decreased between males and females, while no differential change in the response rate was observed.

**Conclusions:**

Providing insights on non-response patterns over time is essential to understand whether declines in response rates may have influenced estimated trends in health behaviours. The framework outlined in this study can be used for this purpose. In our example, in spite of a major decline in response rate, there was no evidence that the risk of non-response bias increased over time.

**Supplementary Information:**

The online version contains supplementary material available at 10.1186/s12874-021-01221-0.

## Background

Health surveys provide valuable information on the distribution of disease in a population, health behaviours associated with diseases, and trends over time. Most surveys have experienced major decreases in response rates over the past decades [1–3]. This increases the possibility of selective participation that may bias estimates of prevalence of health behaviours and also associations between determinants and health behaviours. Especially for studying trends, it is important to disentangle an actual change in disease and underlying health behaviours in the entire population from an observed change due to systematic changes in the composition of respondents of the survey over time.

A low response rate in repeated cross-sectional surveys may influence the prevalence estimates of health behaviours in two ways. First, selective participation may occur by sociodemographic subgroups [4, 5]. If a response rate becomes lower among younger compared to older individuals, and younger individuals participate more frequently in sport activities, the observed prevalence estimate of sport activities in the study sample may underestimate sport activities in the entire population. Selective participation between sociodemographic subgroups is often taken into account by calculating weighting factors such that the composition of the study sample mirrors the entire population for known sociodemographic characteristics [6, 7]. Second, selective participation may take place by the outcome of interest, either a health behaviour or a disease [4, 8]. If a response rate is higher among individuals who participate more frequently in sport activities, the observed prevalence estimate of sport activities in the study sample may overestimate sport activities in the entire population. Correcting for the selective participation for outcomes is often not possible given that, in most surveys, the outcome among those who do not respond is unknown. To make it more complex, the combination of both mechanisms of selective participation may also occur, whereby unhealthy younger persons respond less than healthy older persons, resulting in a gross underestimation of the prevalence of disease and overestimation of health behaviour.

A low response rate also plays a role in estimating the associations between sociodemographic determinants and outcomes, albeit in a different way. Selective participation by sociodemographic subgroups will not influence associations between age and sport participation as long as the persons who respond are a random sample. If younger individuals respond less, but those who do respond participate in sport as frequently as individuals who do not respond, the association between age and sport participation is not influenced by the low response rate. However, if selective participation takes place by the outcome of interest, and only the young individuals who participate in sport respond, the observed estimate for the association between age and sport participation may be overestimated. In addition, at this point a combination of the two selection processes may occur; however, only selective participation by the outcome will bias associations. Correcting for the latter is not possible, although it has been suggested that a low response rate will not bias associations in studies with large samples sizes and sufficient variation in the outcome of interest [9, 10]. This suggestion was supported by a modelling study showing that estimates of associations between determinants and outcomes were relatively unbiased in scenarios where variation was maintained by including some individuals with outcomes at extreme values of the distribution [11].

Bias in estimated trends in health behaviours due to a decreasing - and potentially more selective – number of responses over time can be investigated by comparing the sociodemographic characteristics and outcomes of those who respond to those who do not. Some studies will have complete information of the total sample, for example using information collected in previous studies [4], through linkage with registries on sociodemographic characteristics [5] or databases for specific outcomes such as membership of a sports club [8]. Nevertheless, the majority of outcomes will only be known for those who participate in the survey. Although most health surveys are suffering from a decline in response rates, a step-by-step approach to describe how a decline in responses over time can be explored in repeated cross-sectional surveys is currently lacking.

This study aimed to outline a framework combining multiple analytical strategies to explore the risk of non-response bias over time and to evaluate its usefulness in a population survey where the response rate has considerably decreased over time.

## Methods

### Study population

We used the Rotterdam leisure-time survey *(in Dutch: Vrijetijdsonderzoek)* to obtain data on health behaviours of Rotterdam citizens, the 2nd largest city in The Netherlands with ~ 650,000 inhabitants. This biannual cross-sectional survey was conducted by researchers from the City of Rotterdam from 1995 until 2017. Participants were randomly selected from the municipal registration database and sent a letter of invitation and a questionnaire. The registration database provided information on age, sex, ethnicity, and district of residence, and was linked with questionnaire information on demographics, leisure-time related topics, and a section on sport participation and sedentary behaviours. Respondents were informed that completing the questionnaire implied consent to use the data anonymously for research purposes. The approach to inviting participants and the mode of delivery of data collection changed over time. An overview of the changes is presented in Supplemental Table 1. From 1995 until 2003, a random selection of individuals was invited to participate. Since 2005, specific population subgroups were oversampled to increase the precision of estimates of subgroups with few respondents.

The total study sample for analyses included 33,934 respondents aged 15–75 years for which complete data on age, sex, ethnicity, income, and city district were available. The total study sample for analyses included 25,897 respondents with data on sport participation and 29,615 on watching TV.

### Sport participation and watching TV

The main outcomes of the study were sport participation and watching TV, since these were repeatedly measured with similar questions in at least 10 subsequent biannual surveys. A standardised questionnaire *(in Dutch: Richtlijn Sportdeelname Onderzoek)* was used to ask about sport participation frequency over the past year with the open question: “How many times did you participate in sport over the past 12 months?”, and answers were categorised into weekly (≥46 times/year) or less than weekly (< 46 times/year). Responses on sport participation were available from 1999 to 2017 as the question was asked neither in 1995 nor 1997. Time spent watching TV was asked by the open question: “How many hours, on an average day, do you spend watching TV?”, and responses were categorised into < 3 h/day or ≥ 3 h/day. The cut-off value was based on the results of an earlier study suggesting that watching TV for ≥3 h/day was associated with increased mortality, independent of physical activity [12]. Responses on watching TV were available from 1995 to 2015, but not 2017 as the question was not asked in that year.

### Sociodemographic variables

Information on age, sex, ethnicity, and city district was derived from the municipal registration database. Age was categorised into 16–24, 25–44, 45–64, and 65–75 years. Ethnicity was based on the country of birth and classified as Western and non-Western, following the definition by Statistics Netherlands [13]. Income was obtained from the survey. For each survey, the social minimum income and modal income of that specific year was used as the reference to classify self-reported household income into low (below social minimum), mid-low (social minimum to modal), mid-high (modal to 2x modal), and high (>2x modal) income.

### Statistical analyses

The framework consists of four steps that enable the estimation of whether non-response over time has biased observed trends in prevalence estimates of sport participation and TV watching in the general population and their associations with sociodemographic characteristics. In the first step, we investigated the sociodemographic determinants of responding to each biannual survey, using data from all persons invited to participate in each survey. Logistic regression analysis was conducted with response as the binary outcome variable, and time (survey year), age (four categories), sex (male vs female) and ethnicity (Western vs non-Western) as determinants, using pooled data of all surveys. The odds ratio (OR) estimates the trend in response per two-year interval as the survey was repeated biannually. We tested whether the response over time differed between age groups, sex, and ethnic groups by adding interaction terms with time to the model. Significant interaction terms indicate that the probability of responding for particular age groups, sex, or ethnic groups changed over time.

For the second step, we estimated the variation in outcomes by calculating the mean and standard deviation (SD) in sport participation (times/year) and watching TV (hours/day) in each cross-sectional sample by sociodemographic subgroup. We visualised the patterns in SD over time by age group, sex, and ethnic group.

For the third step, we calculated weighted and unweighted prevalence estimates of sport participation and watching TV in each cross-sectional sample by sociodemographic subgroup. Prevalence estimates were weighted by age, sex, and ethnicity distributions per city district to reflect the Rotterdam population of that year. All weighting variables were obtained from the municipal registration database, and thus available for respondents and non-respondents. The difference between weighted and unweighted prevalence estimates illustrates how the differential response rates between population subgroups affected the prevalence estimates of sport participation and watching TV.

For the fourth step, we estimated associations between sociodemographic determinants and health behaviours. For each cross-sectional sample, two separate logistic regression analyses were conducted with sport participation and watching TV as the binary dependent variable, respectively. Independent variables were age (four age categories), sex (male vs female), and ethnicity (Western vs non-Western). Studies have shown that higher socioeconomic subgroups more often participate in sport activities and spend less time watching TV [14–16], therefore models were additionally adjusted for household income (four income categories). The OR estimates per wave were analysed for changes over time, indicating whether particular age groups, sex, or ethnic groups reported increasing or decreasing sport participation or TV watching over time.

We presented a checklist to summarise whether differential non-response over time may have biased estimated trends in health behaviours. All analyses were conducted in SPSS version 24. Two-sided *P*-values of < 0.05 were considered statistically significant. Interactions were explored for P-for-interactions of < 0.10.

## Results

### Step 1: Sociodemographic determinants of responding to the survey

During 12 waves, a total of 136,696 subjects were invited to participate, and 35,587 subjects returned the questionnaire. The overall response rate declined from 47% in 1995 to 15% in 2017. The characteristics of the study sample are presented in Supplemental Table 2. Over the whole period, the probability of responding was higher among older people, females, and those with a Western background (Table 1).

**Table 1:** Sociodemographic determinants of response to a health survey

	Response OR (95% CI)
Time (per 2 years)	0.92 (0.92; 0.92)
Age (years)	
16–24	1.01 (0.97; 1.05)
25–44 (ref)	1.0
45–64	1.29 (1.25; 1.33)
65–75	1.54 (1.48; 1.61)
Sex	
Male (ref)	1.0
Female	1.29 (1.26; 1.32)
Ethnicity	
Western (ref)	1.0
Non-Western	0.51 (0.49; 0.52)

Results were obtained from a logistic regression model using response to the health survey as a binary outcome, including time as a continuous variable (per 2 years), and sociodemographic determinants as listed. All determinants were derived from the municipal registration database, and thus available for respondents and non-respondents

The average decline in response was 8% every 2 years. Over a 22-year period, this accumulated to a relative decrease of 62% in survey responses. In the first survey conducted in 1995, the overall response rate was 47%, and the absolute difference between the most and least responsive subgroup was 9%-points (Supplemental Table 3). For the most recent survey conducted in 2017, the overall response rate was 15%, and the absolute difference between the most and least responsive subgroup was 15%-points (Supplemental Table 3).

Between 1995 and 2017, the response rate declined for all groups. The changes in response over time differed for age groups and ethnic groups (P-for-interaction< 0.10) but not for sex. The probability of responding decreased by 10% every 2 years for individuals aged 16–24 years, as compared to 4% for individuals aged 65–75 years (Table 2). Over a 22-year period, this accumulated to a 70% decrease in response for individuals aged 16–24 years and a 36% decrease in response for individuals aged 65–75 years. The probability of responding decreased by 7% per 2 years (57% over 22 years) for individuals with a Western background as compared to 10% (69% over 22 years) for individuals with a non-Western background.

**Table 2:** Sociodemographic determinants of the change in response to a health survey between 1995 and 2017

	Change in response per 2 years OR (95% CI)
Age (years)	
16–24	0.90 (0.90; 0.91)
25–44	0.91 (0.91; 0.91)
45–64	0.93 (0.93; 0.94)
65–75	0.96 (0.95; 0.97)
Sex	
Male	0.92 (0.92; 0.92)
Female	0.92 (0.92; 0.92)
Ethnicity	
Western	0.93 (0.93; 0.93)
Non-Western	0.90 (0.90; 0.91)

Results were obtained from a stratified logistic regression model using response to the health survey as a binary outcome, including time as a continuous variable (per 2 years), and sociodemographic determinants as listed. All determinants were derived from the municipal registration database, and thus available for respondents and non-respondents

### Step 2: variation in outcomes over time

**Fig. 1:**
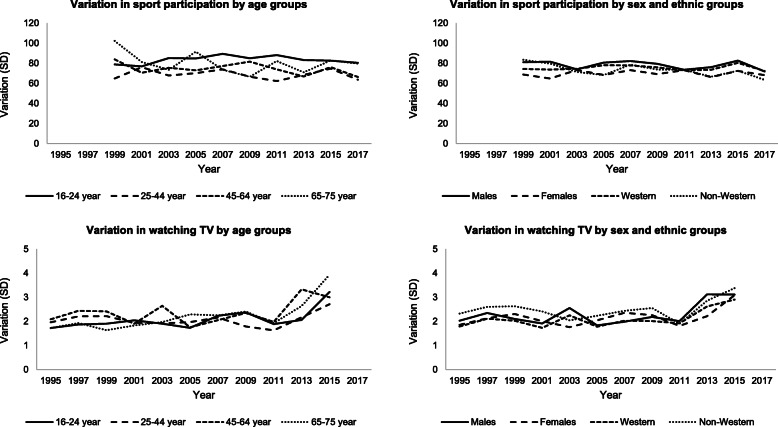
Variation (SD) in sport participation and watching TV by sociodemographic determinants in each cross-sectional sample. Abbreviations: SD = standard deviation

Between 1995 and 2017, the variation in sport participation remained fairly stable across sociodemographic groups (Fig. 1). In the total population, the variation changed from a SD of 76 times/year in 1999 to 70 times/year in 2017. The variation in TV watching increased in the last three rounds of data collection, from a SD of 1.9 h/day in 2011 to 3.1 h/day in 2015. However, there were no large differences between sociodemographic subgroups. No differences in variance were seen in the period that less-responsive subgroups were oversampled as compared to the situation before in which a random selection was invited to participate.

### Step 3: comparison of weighted and unweighted prevalence estimates

**Fig. 2:**
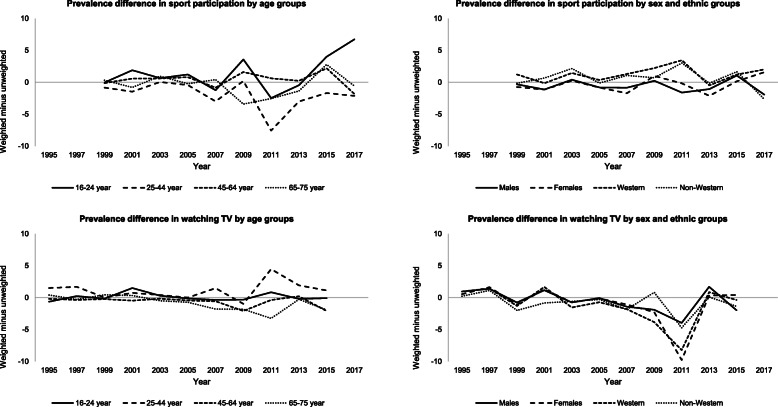
Weighted minus unweighted prevalence estimates (%-points) in sport participation and watching TV by sociodemographic determinants in each cross-sectional sample. Prevalence estimates were weighted for age, sex, and ethnicity distributions per city district to reflect the population of that year

The difference between weighted and unweighted prevalence estimates for sport participation was small for most comparisons in the cross-sectional samples [mean difference: 0.0%-points (interquartile range (IQR): − 1.0–1.1)]. Likewise, weighted and unweighted prevalence estimates for watching TV did not differ largely [mean difference: − 0.5%-points (IQR: − 1.1–0.5)]. There was no clear trend across sociodemographic subgroups (Fig. 2).

### Step 4: comparisons of associations between health behaviours and sociodemographic groups

**Fig. 3:**
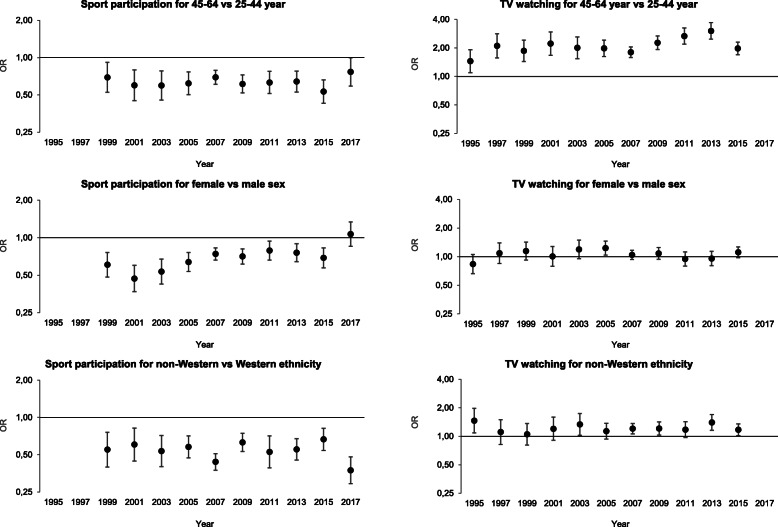
Associations between health behaviours and sociodemographic determinants in each cross-sectional sample. Results were obtained from a logistic regression model using sport participation and watching TV as a binary outcome, and adjusted for age, sex, ethnicity and household income. Abbreviations: OR = odds ratio

In all years, the probability of participating in sport was lower for individuals aged 45–64 years and with a non-Western background as compared to those aged 25–44 years and with a Western background (Fig. 3). The probability of participating in sport was lower for females in earlier years, but over time the difference in sport participation between males and females gradually decreased. The probability of watching TV was higher for individuals aged 45–64 years and those with a non-Western background as compared to those aged 25–44 years and with a Western background. No differences were seen between males and females.

### Summary of the findings

A summary of the findings is presented in Table 3. In spite of a faster decline in response among persons aged 25–44 years and those with a non-Western ethnicity as compared to persons aged 45–64 years and those with a Western ethnicity, the variation in sport participation and watching TV in the study sample remained constant over time. Weighted and unweighted prevalence estimates and associations did not follow the changes in response over time. On the contrary, we observed that the difference in probability of participating in sport gradually decreased between males and females, while the change in response and variation in sport participation in the study sample did not differ over time between males and females. We did not find evidence that the risk of non-response bias increased over time.

**Table 3:** Checklist for the evaluation of non-response over time in repeated cross-sectional studies

Health behaviour as outcome		Sport participation			Watching TV		
Sociodemographic subgroup		Age	Sex	Ethnicity	Age	Sex	Ethnicity
Step 1	Did the change in response over time differ between subgroups?	Y	N	Y	Y	N	Y
Step 2	Did the variation in outcome change over time?	N	N	N	N	N	N
Step 3	Did the difference between weighted and unweighted prevalence estimates of the outcome change over time?	N	N	N	N	N	N
Step 4	Did the association between sociodemographic determinants and outcome change over time?	N	Y	N	N	N	N
Summary	Is it likely that increasing non-response over time biased estimated time trends in outcomes?	N	N	N	N	N	N

## Discussion

We outlined a four-step approach to obtain insights in differential non-response patterns over time, and the influence on estimations of prevalence in health behaviours and associations between sociodemographic characteristics and health behaviours. We applied this framework to the Rotterdam leisure-time survey, a repeated cross-sectional study in which the overall response rate declined from 47% in 1995 to 15% in 2017. We observed that the response rate declined more rapidly among younger participants and those with a non-Western ethnicity. The results did not suggest that the differential non-response biased estimated trends in prevalence of sport participation and watching TV and associations between sociodemographic determinants and sport participation and watching TV.

Health surveys are frequently used by national and local institutions to gain insights on the health of the population at a specific point in time and to evaluate trends over time. It is important to thoroughly examine potential non-response bias in questionnaire surveys. We suggest to report the change in response for each subgroup to provide insights on the risk of bias in some subgroups as compared to others. Whereas most health surveys experienced major decreases in response rates over time, only few have described the presence of differential non-response and its impact on reported findings.

Two studies have previously evaluated the decline in response across subgroups. Responses to a Finnish health survey between 1978 and 2002 declined faster among younger participants and males, and authors suggested that this may have resulted in biased trend estimates [5]. In a Lithuanian study, the decrease in response between 1994 and 2010 was similar in all age groups and between males and females, and authors concluded that this could not seriously bias trends in smoking behaviour [17]. One study evaluated trends in smoking behaviour in 17 populations, using information from persons who immediately returned the questionnaire, and compared this with information collected for part of the population who initially did not respond [18]. They showed that the decreasing trend in smoking prevalence among males was overestimated in most populations when calculating trends only based on data from respondents who immediately handed in the survey. For females, however, the decreasing trend was overestimated in half of the population and underestimated in the other half. Some studies evaluating trends in health behaviours have stated that their findings may be biased due to a larger decline in response among some groups compared to others [5, 8, 19–23]. Most studies, however, did not give further detail on the differences in non-response over time [8, 19–23], therefore judging whether this poses a serious risk is not possible. The framework proposed in this study may be useful for this purpose.

The percentage of people who respond to a survey is often used as the first indicator of potential non-response bias. Tools for the evaluation of methodological quality often classify studies that have a response below 60% as studies with a high risk of bias. However, there is no scientific evidence that supports this cut-off [24]. This classification can be too simplistic, especially when sampling strategies are used whereby people from less-responsive subgroups are more frequently invited. Inviting more people from less-responsive subgroups will directly result in a lower overall response, however, this does not necessarily introduce bias. On the contrary, a modelling study showed that by including at least some persons in upper and lower percentiles of the distributions of outcomes reduced the risk of bias in associations between determinants and outcomes [11]. Recruiting persons from the extremes of the distribution likely increases the variation in the study sample. Following this rationale, we would expect a larger variation in the outcomes from 2005 onwards, but the variation remained fairly constant. However, we do not know what the variation would have been in the years after 2005 without the oversampling strategy. It is likely that the sampling methodology contributed to the relatively high variation in the study sample throughout the study period. When using a sampling strategy, it remains important to invite persons within oversampled subgroups randomly and to capture information that allows for the correct weighting of the study sample back to the population that they represent. Oversampling may come at a cost of small losses in precision for other subgroups and the total population when the total number of questionnaires is fixed [25]. Recruiting and retaining individuals from less-responsive subgroups at the end of the distributions of relevant outcomes is warranted to obtain precise prevalence estimates and lower the risk of bias for associations between determinants and outcomes.

### Strengths and limitations

A strength of this study was the long time series of repeated surveys, which enabled the application of the developed framework to an existing survey that experienced large reductions in response over a 22-year period. The possibility to link both respondents and non-respondents to the municipal registration database provided the unique opportunity to analyse changes in the characteristics of non-respondents over time. An identical set of questions was used to assess sport participation and watching TV in all years. Datasets were harmonised and a weighting variable per survey year was computed using similar methodology. The framework presented in this paper can be used retrospectively to evaluate whether differential non-response has biased trends for various types of outcomes. No additional data needs to be collected.

Our study also has some limitations. The framework depends on the availability of sociodemographic characteristics for respondents and non-respondents and outcomes obtained from the survey. Differential non-response may have occurred for sociodemographic characteristics that remain unknown. Recent developments in linkage with registries, including taxation office and educational achievements, may allow to further detail non-response between sociodemographic subgroups and by outcomes in future studies. Another limitation is that the framework can only be used to evaluate whether the risk of non-response bias has changed over time. Especially for studies with low response rates in early years, the framework does not enable the evaluation of whether estimates in each cross-sectional sample are unbiased. The framework was applied to a population survey in which several aspects around the data collection have changed, possibly influencing the number of responses. In 2011, the respondents could choose to complete the survey digitally or take the pen and paper version. In the same year, door-to-door interviews to recruit non-Dutch participants were discontinued, and the survey was divided into two shorter surveys. We were unable to attribute these multiple changes at the same time to differences in response and whether response rates in some subgroups was more affected than in others.

### Future recommendations

It is important to thoroughly examine potential non-response bias when studying trends in health behaviours. Therefore, we suggest to report response by sociodemographic subgroups. The framework outlined in this paper starts with presenting the differential change in non-response, and from there insights are obtained in bias in estimated trends of health behaviours over time. Recruiting persons from less-responsive subgroups is needed to ensure substantial variation in outcomes in the study sample. The framework can be applied to surveys that collect data on at least two demographic subgroups and two outcome variables at multiple time-points. This allows for the exploration of whether trends in the two outcomes follow the patterns in non-response across these subgroups. A larger number of demographic subgroups and outcome variables will strengthen the conclusion on likelihood of bias in the trends in outcomes due to differential non-response.

## Conclusions

Surveys are an important source of information about the health of a population. A decreasing - and potentially more selective - response over time could increase the risk of bias in studies investigating trends. We provided a framework to assess whether non-response patterns over time resulted in biased trends in health behaviours. The framework was applied to a survey in which response rates gradually decreased over time. In spite of a major decline in overall response rates, and a faster decline in response in some subgroups than others, we did not find evidence that the risk of bias due to non-response has increased over time. Our results suggests that ensuring substantial variation in outcomes rather than ensuring a high response rate is important to reduce the risk of bias.

## Supplementary Information

**Additional file 1: Appendix 1.** Questionnaire items. **Table S1.** Description of the approach to invite participants and the data collection procedures from 1995 to 2017. **Table S2.** Population characteristics from 1995 to 2017. **Table S3.** Response rate in the total population and by age group, sex and ethnic group.

## Data Availability

The data that support the findings of this study are available from the City of Rotterdam, but restrictions apply to the availability of these data, which were used under license for the current study, and are not publicly available. Data are however available from the authors upon reasonable request and with permission of the City of Rotterdam.

## Abbreviations

OR: Odds ratio
SD: Standard deviation
