# Supplementary material for: A framework for exploring non-response patterns over time in health surveys
Source: BMC Med Res Methodol. 2021 Feb 18;21:37. doi: 10.1186/s12874-021-01221-0 (PMC7890886; doi:10.1186/s12874-021-01221-0)
Supplement: Supplementary file 1 — Additional file 1: Appendix 1. Questionnaire items. Table S1. Description of the approach to invite participants and the data collection procedures from 1995 to 2017. Table S2. Population characteristics from 1995 to 2017. Table S3. Response rate in the total population and by age group, sex and ethnic group. [file 12874_2021_1221_MOESM1_ESM.docx]

**Online Supplemental Material**

Article: A framework for exploring non-response patterns over time in health surveys

Authors: Famke J.M. Mölenberg, Chris de Vries, Alex Burdorf, Frank J. van Lenthe

Contents:

Appendix 1 Questionnaire items

Supplemental Table 1 Description of the approach to invite participants and the data collection procedures from 1995 to 2017

Supplemental Table 2 Population characteristics from 1995 to 2017

Supplemental Table 3 Response rate in the total population and by age group, sex and ethnic group

**Appendix 1 Questionnaire items**

The following questions were used to collect information on the main outcomes sport participation and watching TV. These outcomes were repeatedly measured with similar questions in at least 10 subsequent biannual surveys. The translated English language version and the original Dutch language version are given.

**Sport participation:**


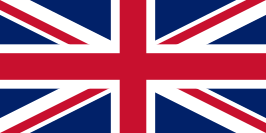
 **How many times did you participate in sport over the past 12 months *in total*?**

*If you frequently participated in sports, please make an estimation of the number of times.*

times

*
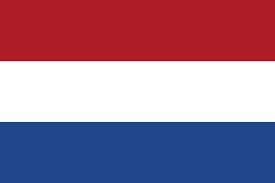
* **Hoeveel keer heeft u in de afgelopen twaalf maanden *in totaal* gesport?**

*Als u veel aan sport heeft gedaan, maak dan een schatting van het aantal keer.*

keer

**Watching TV:**


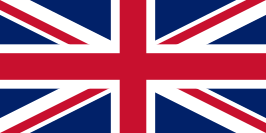
 **How many hours, on an average day, do you spend watching TV?**

hours per day

*
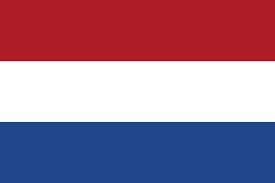
* **Hoeveel uur kijkt u gemiddeld per dag televisie?**

uur per dag

**Supplemental Table 1**. Description of the approach to invite participants and the data collection procedures from 1995 to 2017

|  | 1995 | 1997 | 1999 | 2001 | 2003 | 2005 | 2007 | 2009 | 2011 | 2013 | 2015 | 2017 |
| --- | --- | --- | --- | --- | --- | --- | --- | --- | --- | --- | --- | --- |
| Pen and paper survey | X | X | X | X | X | X | X | X |  |  |  |  |
| Digital survey |  |  |  |  |  |  |  | X | X | X | X | X |
|  |  |  |  |  |  |  |  |  |  |  |  |  |
| Additional door-to-door interviews to recruit non-Dutch participants | X | X | X | X | X | X | X | X |  |  |  |  |
| No additional door-to-door interviews |  |  |  |  |  |  |  |  | X | X | X | X |
|  |  |  |  |  |  |  |  |  |  |  |  |  |
| Random selection of participants | X | X | X | X | X |  |  |  |  |  |  |  |
| Stratified by city district and oversampling of less-responsive subgroups |  |  |  |  |  | X | X | X | X | X | X | X |
| Oversampling of elderly aged 55-75 years |  |  |  |  |  |  | X |  |  |  |  |  |
|  |  |  |  |  |  |  |  |  |  |  |  |  |
| Single survey with all items | X | X | X | X | X | X | X | X |  | X |  | X |
| Two surveys with half of the items |  |  |  |  |  |  |  |  | X |  | X |  |

**Supplemental Table 2.** Population characteristics from 1995 to 2017

|  | | 1995 (n=1404) | 1997 (n=1285) | 1999 (n=1610) | 2001 (n=1270) | 2003 (n=1473) | 2005 (n=2603) | 2007 (n=5966) | 2009 (n=4091) | 2011 (n=5207) | 2013 (n=2743) | 2015 (n=4705) | 2017 (n=1577) |
| --- | --- | --- | --- | --- | --- | --- | --- | --- | --- | --- | --- | --- | --- |
| Age, years (%) | |  |  |  |  |  |  |  |  |  |  |  |  |
|  | 16-24 | 16,7 | 15,7 | 15,7 | 15,9 | 16,6 | 16,6 | 16,6 | 16,7 | 16,5 | 16,5 | 16,4 | 16,3 |
|  | 25-44 | 43,8 | 44,2 | 44,4 | 44,2 | 43,3 | 42,9 | 41,7 | 41,0 | 40,7 | 40,3 | 39,9 | 39,9 |
|  | 45-64 | 27,5 | 28,1 | 28,5 | 28,8 | 29,4 | 30,0 | 31,3 | 32,0 | 32,7 | 32,2 | 32,2 | 32,3 |
|  | 65-75 | 12,0 | 12,0 | 11,4 | 11,0 | 10,6 | 10,5 | 10,5 | 10,3 | 10,1 | 11,0 | 11,5 | 11,5 |
| Sex (%) | |  |  |  |  |  |  |  |  |  |  |  |  |
|  | Male | 50,0 | 50,0 | 50,0 | 50,0 | 50,0 | 50,0 | 50,0 | 50,0 | 50,0 | 50,0 | 50,0 | 50,0 |
|  | Female | 50,0 | 50,0 | 50,0 | 50,0 | 50,0 | 50,0 | 50,0 | 50,0 | 50,0 | 50,0 | 50,0 | 50,0 |
| Ethnicity (%) | |  |  |  |  |  |  |  |  |  |  |  |  |
|  | Western | 77,3 | 75,7 | 73,5 | 70,6 | 68,8 | 67,1 | 66,0 | 64,7 | 63,7 | 62,7 | 61,8 | 61,0 |
|  | Non-Western | 22,7 | 24,3 | 26,5 | 29,4 | 31,2 | 32,9 | 34,0 | 35,3 | 36,3 | 37,3 | 38,2 | 39,0 |
| Household income (%) | |  |  |  |  |  |  |  |  |  |  |  |  |
|  | Low | 23,8 | 20,5 | 19,9 | 21,8 | 22,4 | 23,6 | 22,4 | 23,6 | 25,8 | 34,5 | 31,5 | 26,3 |
|  | Mid-low | 24,6 | 27,0 | 25,7 | 27,8 | 29,3 | 28,3 | 28,1 | 22,5 | 21,0 | 20,5 | 21,5 | 24,4 |
|  | Mid-high | 36,1 | 37,3 | 36,9 | 34,0 | 27,2 | 28,6 | 28,0 | 31,3 | 28,7 | 26,4 | 25,1 | 25,7 |
|  | High | 15,5 | 15,3 | 17,5 | 16,4 | 21,1 | 19,5 | 21,5 | 22,5 | 24,4 | 18,6 | 22,0 | 23,6 |
| Sport participation (%) | |  |  |  |  |  |  |  |  |  |  |  |  |
|  | Less than weekly | NA^a^ | NA^a^ | 66,0 | 61,4 | 61,3 | 62,9 | 62,2 | 58,9 | 56,8 | 59,6 | 51,0 | 58,0 |
|  | Weekly | NA^a^ | NA^a^ | 34,0 | 38,6 | 38,7 | 37,1 | 37,8 | 41,1 | 43,2 | 40,4 | 49,0 | 42,0 |
| Watching TV (%) | |  |  |  |  |  |  |  |  |  |  |  |  |
|  | <3 hours/day | 44,8 | 50,6 | 51,9 | 48,7 | 52,9 | 54,0 | 56,5 | 56,1 | 57,5 | 60,0 | 61,7 | NA^b^ |
|  | ≥3 hours/day | 55,2 | 49,4 | 48,1 | 51,3 | 47,1 | 46,0 | 43,5 | 43,9 | 42,5 | 40,0 | 38,3 | NA^b^ |

Prevalence estimates were weighted for age, sex, and ethnicity distributions per city district to reflect the population of that year.
^a^ Responses on sport participation were available from 1999 to 2017.
^b^ Responses on watching TV were available from 1995 to 2015.

**Supplemental Table 3**. Response rate in the total population and by age group, sex and ethnic group

|  | 1995 | 1997 | 1999 | 2001 | 2003 | 2005 | 2007 | 2009 | 2011 | 2013 | 2015 | 2017 |
| --- | --- | --- | --- | --- | --- | --- | --- | --- | --- | --- | --- | --- |
| Overall response (%) | 46,8 | 43,5 | 54,0 | 48,6 | 53,8 | 42,8 | 36,7 | 20,1 | 33,1 | 23,0 | 20,5 | 14,8 |
| Age, years (%) |  |  |  |  |  |  |  |  |  |  |  |  |
| 16-24 | 44,8 | 47,0 | 52,8 | 46,2 | 52,1 | 38,4 | 29,8 | 18,3 | 25,0 | 19,0 | 17,5 | 12,3 |
| 25-44 | 48,2 | 43,4 | 53,1 | 47,3 | 50,2 | 40,7 | 33,7 | 17,7 | 26,3 | 20,9 | 17,7 | 12,5 |
| 45-64 | 48,1 | 42,9 | 53,4 | 51,5 | 57,6 | 47,9 | 41,0 | 23,0 | 36,5 | 26,0 | 24,0 | 17,2 |
| 65-75 | 41,7 | 40,9 | 59,9 | 49,2 | 59,3 | 53,3 | 44,3 | 24,5 | 46,6 | 34,6 | 30,9 | 25,1 |
| Sex (%) |  |  |  |  |  |  |  |  |  |  |  |  |
| Male | 42,3 | 42,1 | 50,3 | 44,8 | 50,5 | 37,9 | 34,0 | 18,5 | 29,8 | 21,2 | 18,4 | 13,8 |
| Female | 50,5 | 44,9 | 57,5 | 52,2 | 57,3 | 47,6 | 39,4 | 21,7 | 36,2 | 24,8 | 22,6 | 15,8 |
| Ethnicity (%) |  |  |  |  |  |  |  |  |  |  |  |  |
| Western | 47,0 | 45,3 | 57,8 | 52,4 | 56,5 | 49,9 | 40,8 | 24,8 | 36,9 | 30,3 | 27,3 | 20,1 |
| Non-Western | 46,3 | 38,1 | 43,3 | 40,1 | 48,0 | 32,7 | 27,6 | 14,0 | 20,2 | 15,1 | 14,5 | 10,3 |
